# The Edinburgh Medical School

**Published:** 1910-03-12

**Authors:** 


### The Edinburgh Medical School.

That the authorities of the Edinburgh schools have been and are in earnest in their efforts to improve and reform the teaching and training which is offered in the Scottish capital to students of medicine has been made fully evident during the past year. Some ten months ago (The Hospital, May 29, 1909, p. 223) the scheme put forward by Dr. Byrom Bramwell, in response to the invitation of the Edinburgh University Pathological Club, was summarised and discussed. Since then various reforms and changes have been adopted, and already the results of them are in some cases ripe for further comment. In certain respects it would seem that experience has brought to light defects in the new system, and it is said that the University authorities are already revolving plans for the further amendment of the rearranged curriculum.

In Dr. Bramwell's report stress was laid upon the water-tight compartment character of some of the examinations, whereby students had been encouraged to concentrate far too much upon one subject at a time, and far too much upon theoretical, as opposed to practical, clinical work. This tendency the new regulations have not succeeded in overcoming, but it is satisfactory to see that its disadvantages are receiving recognition, and that the expected further instalments of reform are likely to include measures calculated to promote its decay*. Too frequent recurrence of examinations is another complaint that has been raised; sessions are interrupted midway by some examination which destroys the value of whole lecture courses. Thus the examination in physics leads to the neglect of chemistry all through the first half of the winter session, and that in anatomy similarly interrupts the systematic courses on pathology and surgery. It would appear from this latter complaint that the old system of starting clinical work before passing in the preliminary and ancillary sciences is still, to some extent, maintained; probably before long the wisdom will be recognised of postponing the ward work until undivided attention can be given to the strictly professional side of the medical student's training.

Broadly, the editorial comment of the journal quoted is, that the reforms are not sufficiently comprehensive, and that not all of them have proved workable; further instalments, but not too hastily settled or too scantily considered, are deemed imperative. An interesting sidelight upon the medical teaching at Edinburgh, as it was ten years ago, is contributed† to the *St. Mary's Hospital Gazette* by Mr. D. C. L. Fitzwilliams, an old Edinburgh man, who has recently joined the surgical staff at that hospital. The author is too wary to be drawn into any comparison of London with Scottish methods of teaching, but he has much that is interesting to say of the old *régime* which the Edinburgh reformers are now endeavouring to sweep away. He shows how the unfortunate student had 1,500 formal lectures to attend, not counting demonstrations and practical work. The old order dies hard, and even Dr. Bramwell ventured to propose but a moderate reduction of this inordinate number. Probably the system of internal examiners had something to do with the survival of the lecture and the comparative neglect of the text-book; for Mr. Fitzwilliams assures us that nearly all the students took most voluminous notes, which they read in preference to text-books. The co-existent neglect of clinical study is similarly accounted for by the very limited supply of out-patient cases at the hospitals. No doubt the evolution of the text-book and its lessening cost will drive one more nail in the coffin of the signing-up system, whose days must surely be numbered, even in Edinburgh. The further steps which are to be taken for improving clinical teaching and maintaining worthily the splendid standard of the past will be awaited with interest on this side of the Tweed, and the results watched with close attention.

* *Edinburgh Medical Journal*, January 1910., pp. 2-3.
† *St. Mary's Hospital Gazette*, February 1910.

